# High-Throughput and Accurate 3D Scanning of Cattle Using Time-of-Flight Sensors and Deep Learning

**DOI:** 10.3390/s24165275

**Published:** 2024-08-14

**Authors:** Gbenga Omotara, Seyed Mohamad Ali Tousi, Jared Decker, Derek Brake, G. N. DeSouza

**Affiliations:** 1 Vision-Guided and Intelligent Robotics Laboratory, Electrical Engineering and Computer Science Department, University of Missouri, Columbia, MO 65201, USA; goowfd@mail.missouri.edu (G.O.); stousi@mail.missouri.edu (S.M.A.T.); 2 Division of Animal Sciences, University of Missouri, Columbia, MO 65201, USA; deckerje@missouri.edu (J.D.); braked@missouri.edu (D.B.)

**Keywords:** cattle scanner, deep learning, segmentation, 3D surface reconstruction

## Abstract

We introduce a high-throughput 3D scanning system designed to accurately measure cattle phenotypes. This scanner employs an array of depth sensors, i.e., time-of-flight (ToF) sensors, each controlled by dedicated embedded devices. The sensors generate high-fidelity 3D point clouds, which are automatically *stitched* using a point could segmentation approach through deep learning. The deep learner combines raw RGB and depth data to identify correspondences between the multiple 3D point clouds, thus creating a single and accurate mesh that reconstructs the cattle geometry on the fly. In order to evaluate the performance of our system, we implemented a two-fold validation process. Initially, we quantitatively tested the scanner for its ability to determine accurate volume and surface area measurements in a controlled environment featuring known objects. Next, we explored the impact and need for multi-device synchronization when scanning moving targets (cattle). Finally, we performed qualitative and quantitative measurements on cattle. The experimental results demonstrate that the proposed system is capable of producing high-quality meshes of untamed cattle with accurate volume and surface area measurements for livestock studies.

## 1. Introduction

On 15 November 2022, the world’s population surpassed the milestone of 8 billion people [1], thereby accentuating the significance of food security on a global scale. The reliable quantification of livestock and plant phenotypes has emerged as a vital factor in maximizing efficiency and sustainability in food production.

Cattle farming forms an integral part of global agriculture, serving as a substantial contributor to food resources and economic stability for numerous communities. In the wake of technological advancements, we see an increasing adoption of innovative practices aimed at enhancing productivity and sustainability in cattle farming. One such technological breakthrough proposed here is the automated extraction of cattle phenotypes through 3D cattle scanning.

Utilizing 3D scanning to capture the volume and surface area of cattle enables farmers and researchers to extract valuable insights about the animals’ health, growth trends, and overall welfare [2]. These outcomes are possible because 3D scanning facilitates accurate and continuous body condition scoring, which supports optimized feed and nutrition management strategies.

There are several significant challenges in the field of 3D cattle scanning that must be overcome to develop a reliable and accurate system capable of delivering the aforementioned benefits. Here are some of the most critical issues:

1. **Untamed Animals**: Scanning cattle managed on pastures or rangelands which rarely interact with humans presents a considerable challenge due to their unpredictable movements. Restraint using firm structures is necessary, but these structures complicate the 3D reconstruction process as they must be segmented out from the captured model, adding complexity and potential for errors.
2. **Synchronization of Capturing Process**: A robust and meticulously designed synchronization process is essential to minimize errors during the capture phase, particularly with untamed animals. Inadequate synchronization can lead to inaccurate data due to animal movement during scanning.
3. **Need for Manual Management of Captured Data**: The requirement for manual segmentation, reconstruction, and camera calibration for each scan impedes the development of a high-throughput system. This manual intervention slows down the process, making it challenging to scan a large number of animals quickly and efficiently.

Addressing these challenges is crucial for advancing the technology and enhancing its practical application in livestock management.

Here, we introduce a 3D cattle scanning system designed to overcome the aforementioned challenges. Our system employs RGBD cameras (Microsoft Corporation, Redmond, WA, USA) to generate precise and reliable 3D models of cattle, automating the process of calculating cattle volume and surface area for high-throughput applications. The structure of this paper is as follows: Section 2 reviews previous work in the areas of 3D animal scanning, 3D point cloud registration, and 3D surface reconstruction. It establishes the context for our innovations and the need for advancements over existing technologies. In Section 3, we provide a detailed overview of our proposed scanning system, describing the technologies and methodologies incorporated into its pipeline. We also compare our system’s capabilities with those of existing solutions, highlighting the improvements and advantages our system offers. In Section 4, we detail the experiments conducted to test the efficacy of our scanning system. We discuss the setup, execution, and outcomes of these experiments, providing empirical evidence of the system’s performance. Section 5 is the conclusion with a comprehensive discussion of the experimental results, evaluating the system’s effectiveness in various operational scenarios. We also discuss the broader implications of our findings, the system’s potential impact, and directions for future research and development.

## 2. Background and Related Work

The domain of computer vision in measuring livestock physical traits is experiencing rapid growth, as evidenced by several impactful studies. For instance, the research discussed in [3] employed three Kinect V1 sensors to evaluate the quality of Japanese Black cattle. This system benefited from the inclusion of a single tilt sensor that helped adjust the orientation of the animals post data collection. However, this system has limitations, such as its inability to scan multiple animals quickly due to its manual operation. Additionally, it employs a calibration and scanning technique that is not robust enough for scanning untrained animals.

Another study [4] addressed the issue of measurement accuracy by using a 3D thermo-sensing device in conjunction with a handheld 3D scanner, which eliminates the need for camera calibration to construct 3D models. Although the results were promising when tested on known objects to verify accuracy, the study did not extend these findings to actual cattle, leaving questions about the accuracy and completeness of the 3D models of live subjects. Furthermore, the throughput of their system remains low, as it can perform only a limited number of scans within a given time frame.

The study in [5] employs three Kinect cameras, strategically positioned to synchronize views and construct 3D models of cattle. A notable aspect of their approach is the attempt to synchronize the capture process using message passing over a local network. Similarly, the research described in [6] also utilizes software-based synchronization while allowing animals to walk through the scanner. However, both approaches face challenges with software-based synchronization via local networks, which can be unreliable due to network congestion or sudden movements of the animals. Moreover, both methods require animals to traverse the scanning area without restraint. This practice can introduce errors due to animal movement and is also ineffective for scanning untamed animals, limiting the applicability of these techniques in real-world scenarios.

To tackle the issues associated with fixed scanning setups and the difficulties in managing animal movement during scanning, [7] introduces a novel portable scanner. This device employs two scanning cameras mounted on a sturdy, portable frame, simplifying the calibration process due to the fixed nature of the cameras. This design makes the calibration straightforward and less prone to error. However, the study lacks a crucial component: it does not report accuracy tests using known objects, which is essential for validating the precision of the system.

Moreover, the proposed scanning methodology in [7]—where the scanner is mounted on a human operator who then moves around the animals—proves impractical for scanning large numbers of cattle. This approach requires substantial time and effort per animal, making it inefficient for use in large-scale farming operations where quick and numerous scans might be necessary.

### Contributions

We propose a system that expands on the growing body of research in animal phenotyping by presenting a high-throughput sensor rig comprising a flexible number of time-of-flight sensors arranged on a frame. Our scanning system offers three distinct advantages over comparable systems:

1. **Flexibility**: The automatic point cloud registration provides operators with the flexibility to disassemble and reassemble the camera set in any configuration they desire. This versatility facilitates the system’s transport, making it both possible and practical.
2. **High Throughput**: By employing multiple advanced algorithms to process each cattle’s data, such as a deep learning-based segmentation model and an autonomous image point cloud registration method, the system accelerates data acquisition and processing. This allows operators to scan numerous animals within a relatively short time.
3. **Ability to Scan Untamed Animals**: Scanning untamed animals, which are considerably more challenging to restrain and scan, necessitates a robust and narrow chute to keep the animal still during the data capture. This narrow chute features solid horizontal bars that limit the animal’s space and a manually activated *head catch* that securely holds the animal’s neck, preventing any movement. Such narrow chutes can complicate the point cloud segmentation of the animal. However, since the developed software can successfully *segment the animals* from other objects even in *highly cluttered environments*, the frame can be mounted encircling a chute or any other device suitable for containing untamed animals.

We show that our system can produce highly accurate scans, while extracting animal traits in real-time. This efficiency is largely due to the utilization of a sophisticated image segmentation model, which relies on both depth and RGB images to automate the same segmentation process.

## 3. Proposed Scanning System

In this section, we will describe our proposed platform to build the 3D cattle scanner system and its hardware and software implementation details.

### 3.1. Platform

**Figure 1:**
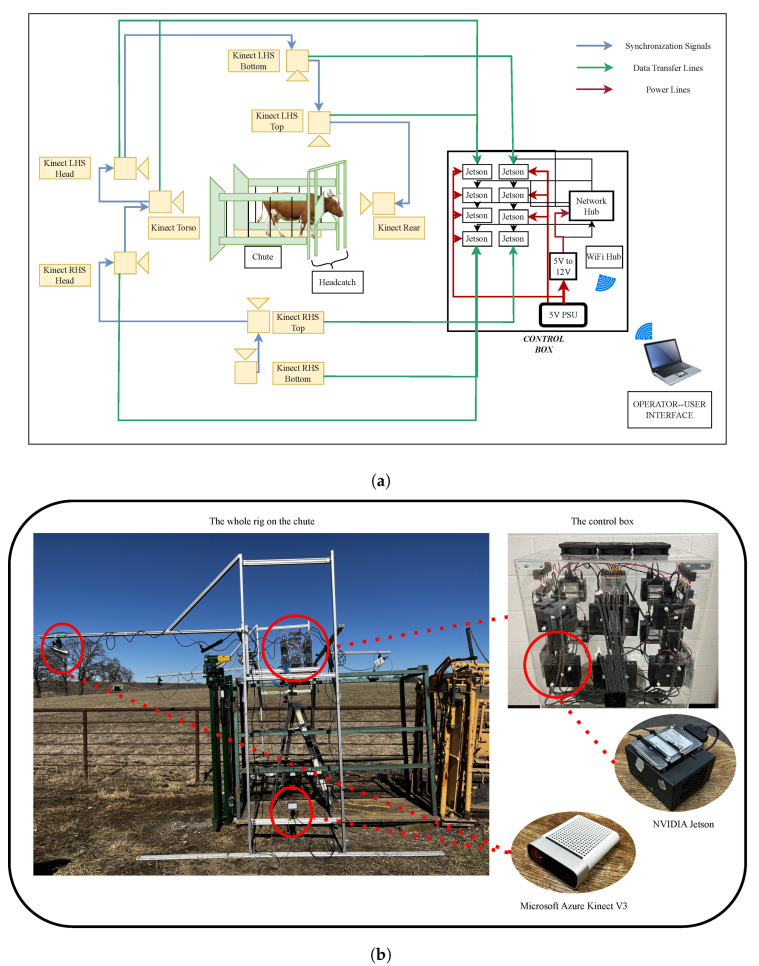
(**a**) A schematic representation of the scanning system. (**b**) Real-life figure of the camera frame and the system components.

Figure 1 presents the platform used in our 3D cattle scanner. The frame is completely isolated from and mounted around the chute to prevent any disturbances due to the animals’ movements. A control box (Figure 1b) is responsible for controlling the cameras and taking images of the cattle which will be described in detail in what follows.

### 3.2. Hardware

The hardware for the scanner consists of three main parts: the camera setup, the control box, and the user interface. The camera setup is flexible and designed to create a volume large enough to cover the entire animal. The current setup for large beef and dairy cattle is as follows:

(a) Two cameras in the front to cover the head/neck of the animal.
(b) Two cameras on top to cover the dorsal view of the animal.
(c) Two cameras on each side to cover the lateral and ventral portions of the animal.

The automatic point cloud registration described in the software section allows for a flexible camera setup that is not tied to any specific arrangement. During our experiments (detailed in Section 4), we varied the setup to handle both small, known objects at close proximity for accuracy and interference measurements, as well as practical applications such as cattle scanning. In each case, the system adapted to the number and spatial arrangement of sensors used.

The control box shows the next component of the scanner system hardware. As depicted in Figure 1, the control box houses the embedded devices (Nvidia Corporation, Santa Clara, CA, USA), a pair of wire networking hubs, a wireless hub, and the power distribution unit. Each embedded device is paired with an individual ToF sensor and manages the image acquisition of its respective camera. A total of up to ten embedded devices and sensors can be installed in one control box, and multiple control boxes can be interconnected for additional ToF sensors. All elements within the control box are linked via a private wireless network to the user interface, enabling control over the scanning procedure.

### 3.3. Software and Pipeline

**Figure 2:**
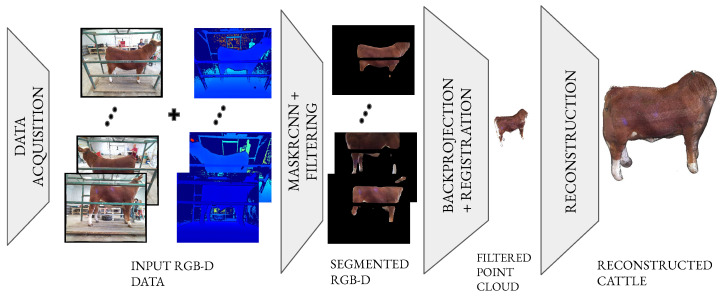
Overview of the software pipeline: The pipeline begins with the data acquisition of RGBD data, which undergo a segmentation and filtering step to eliminate the background pixels and noise in both depth and RGB space. The filtered data are subsequently backprojected into 3D space and then stitched to form a unified 3D model. A mesh is then constructed over the 3D point cloud. Finally, we measure our traits of interest, volume, and surface area.

As depicted in Figure 2, the block diagram outlines the proposed software pipeline of the scanner along with a more detailed summary in Pipelines 1 and 2. The software for the scanning system has four (4) major components: acquisition, processing, registration and trait-extraction.

#### 3.3.1. Acquisition

**Figure 3:**
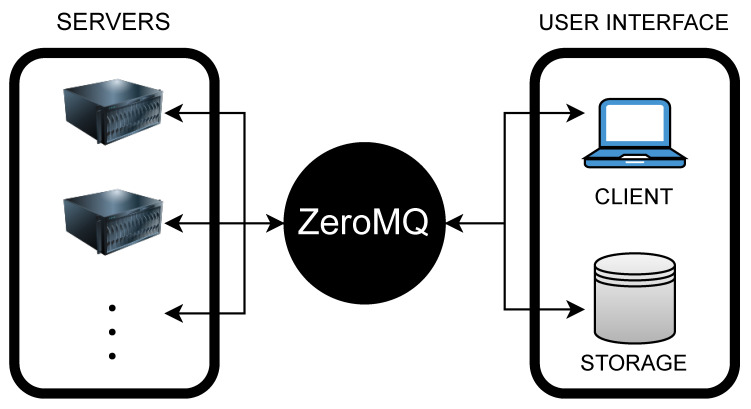
Schematic layout of Server–Client: In this configuration, the Client sends a capture request to 10 Server programs. Each Server program performs the image acquisition request from the Client and the captured data are transmitted to a storage device.

The scanning process starts with the acquisition of RGBD (color and depth) images of the animal, captured by the entire camera assembly. A user-friendly software has been designed, providing operators the ability to customize settings for the image acquisition process, such as an automated or manual assignment of cattle IDs. This software, henceforth known as the Client program, establishes connections with all the embedded devices. Each embedded device hosts a Server program that processes requests from the Client and orchestrates the data acquisition process at a software level. Socket connections facilitated by the ZeroMQ messaging protocol enabled communication between the Client and the Server programs. Following image collection, all RGB and depth images are saved locally on the embedded devices along with each sensor’s intrinsic parameters. These images are then transmitted to the operator’s laptop over a private network for subsequent processing. See Figure 3.
**Pipeline 1** Data Acquisition Protocol for 3D Scanner1:Initialize Client on host device2:Establish ZeroMQ connections to all Servers (embedded devices)3:**for** each Server **do**4:    Send acquisition trigger to Server5:    Wait for acknowledgment from Server6:    Retrieve data from Server7:    Process and store incoming data8:**end for**9:Terminate connections

#### 3.3.2. Processing

In the next step of our pipeline, RGB and depth images are processed to segment the animal’s body, a crucial step for subsequent 3D registration. Initially, we apply a filtering technique based on the method proposed in [8], effectively mitigating the “flying pixel” artifacts typical of ToF sensors.

**Figure 4:**
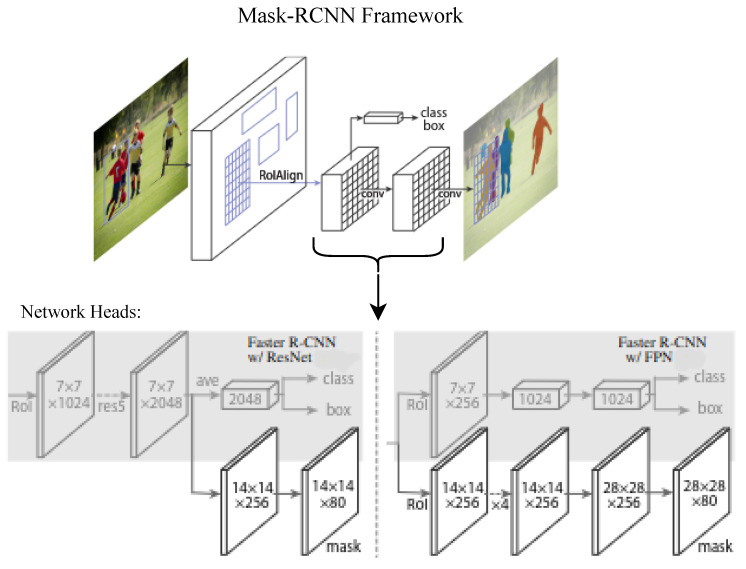
**Mask R-CNN Architecture** [9]: Mask R-CNN builds upon two existing Faster R-CNN heads as detailed in [10,11]. The left and right panels illustrate the heads for the ResNet C4 and FPN backbones, respectively, with an added mask branch. Spatial resolution and channels are indicated by the numbers, while arrows represent conv, deconv, or FC layers, inferred from the context (conv layers maintain spatial dimensions, whereas deconv layers increase them). All conv layers are 3 × 3, except for the output conv which is 1 × 1. Deconv layers are 2 × 2 with a stride of 2, and ReLU [12] is used in hidden layers. On the left, ‘res5’ refers to the fifth stage of ResNet, which has been modified so that the first conv layer operates on a 7 × 7 RoI with a stride of 1 (instead of 14 × 14 with a stride of 2 as in [10]). On the right, ‘×4’ indicates a stack of four consecutive conv layers.

Following this, we employed Mask R-CNN (Region-based Convolutional Neural Network), a state-of-the-art deep learning model renowned for its efficacy in image segmentation [9]. Figure 4 shows a detailed description of the Mask R-CNN architecture.

Our contribution lies in the novel adaptation of the Mask R-CNN framework, which is traditionally trained solely on RGB images from the COCO (common objects in context) dataset. In other words, we expanded the work in [13], by using both RGB and depth inputs and by fine-tuning the Mask R-CNN through a multi-task loss function, we combined three different types of losses: classification loss ($L_{cls}$), bounding box loss ($L_{box}$), and mask loss ($L_{mask}$). The overall loss function is represented as $L=L_{cls}+L_{box}+L_{mask}$. The classification loss given by $L_{cls}=-\sum_{i}\left(y_{i}log\left(pi\right)\right)$ is a cross-entropy loss which measures the difference between the predicted class probabilities and the true class labels for each region of interest, where $y_{i}$ is the ground truth one-hot encoded class label, and $p_{i}$ is the predicted probability of the *i*-th region of interest belonging to the true class. The bounding box loss is a smooth L1 loss which measures the difference between the predicted bounding box coordinates and the ground truth coordinates. It is given by $L_{box}=\sum_{i}smooth_{L1}\left(t_{i}-t_{i}^{*}\right)$, where $t_{i}$ is the predicted bounding box coordinates for the *i*-th ROI, and $t_{i}^{*}$ is the ground truth bounding box coordinates for the *i*-th ROI. And $smooth_{L1}\left(x\right)=0.5x^{2}$ if |x|<1 and |x|−0.5 otherwise. Then, the mask loss is a pixel-wise binary cross-entropy loss applied to the predicted segmentation masks and the ground truth masks given by $L_{mask}=-\sum_{i}(\left[m_{i}log\left(p_{i}\right)+\left(1-m_{i}\right)log\left(1-pi\right)\right]$, where $m_{i}$ is the ground truth mask label for the *i*-th pixel and $p_{i}$ is the predicted probability of the *i*-th pixel being in the object mask.

As indicated above, in the proposed approach, two separate Mask R-CNN networks were fine-tuned on two different input types using the same hand-labeled cattle dataset: RGB and depth. The final mask prediction is obtained by performing an OR operation on the masks generated by the two models. Formally, let $M_{RGB}$ be the mask predicted by the RGB model and $M_{depth}$ be the mask predicted by the depth model. The final mask $M_{final}$ can be obtained as $M_{final}=M_{RGB}\vee M_{depth}$, where ∨ denotes the element-wise logical OR operation. This approach uses the complementary information provided by the RGB and depth images to improve the accuracy and robustness of the final segmentation mask.

The usage of RGB+depth inputs significantly enhances the model’s capacity to accurately segment cattle within our application-specific dataset. By integrating depth data, our fine-tuned Mask R-CNN not only retains its original segmentation capabilities but also gains an increased sensitivity to the spatial nuances critical to effective 3D scanning in agricultural settings.

#### 3.3.3. Registration

**Figure 5:**
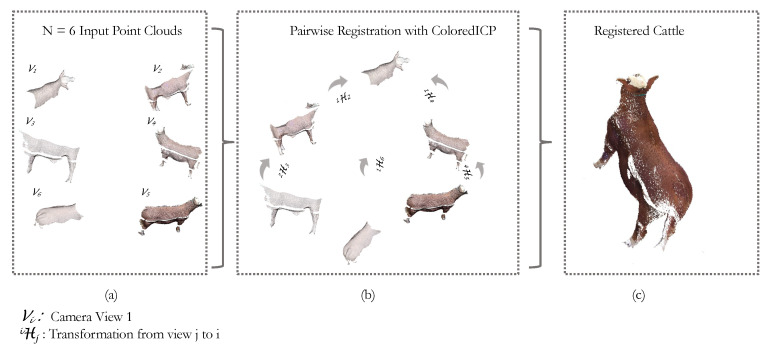
Multi-view point cloud registration: (**a**) Given N = 6 point clouds, we perform a simple pairwise registration of point cloud fragments of the scanned cattle. (**b**) We use the Colored ICP algorithm to solve for the coordinate transformation from camera coordinate frame j to camera coordinate frame i (denoted as ${\;}^{i}H_{j}$). Each view is aligned into the coordinate frame of its adjacent camera. We fix the coordinate frame of Camera 1 ($V_{1}$) as the world coordinate frame and then align all views with respect to coordinate frame 1. (**c**) This results in a well-aligned point cloud of the scanned cattle.

After segmentation, the 2D coordinates of the segmented object are backprojected into 3D space, resulting in a segmented point cloud for each sensor. These point clouds are then stitched/registered to construct a 3D model of the cattle as illustrated in Figure 5.

The task of registering all the point clouds was performed in a pairwise manner, utilizing the multi-scale Colored Iterative Closest Point (ICP) algorithm [14,15] with an initial alignment. The initial alignment of the point clouds was facilitated through the use of a specially designed calibration cube. This cube was embedded with AprilTag fiduciaries [16], a type of 2D barcode, which provides reliable and accurate 3D pose estimation. This initial alignment sets the stage for the more refined multi-scale Colored ICP algorithm. Subsequent to this initial alignment, the point clouds from each pair are registered using the Colored ICP algorithm. The algorithm iteratively minimizes the difference between the points of two point clouds, simultaneously taking into account the color and geometric information. The optimization is performed over the objective function using the Colored ICP as follows:

(1) $$E\left(T\right)=\left(1-\delta \right)E_{C}\left(T\right)+\delta E_{G}\left(T\right)$$

where *T* is the transformation matrix to be estimated; $E_{C}$ and $E_{G}$ are the photometric and geometric terms, respectively; and δ∈[0,1] is a weight parameter that has been determined empirically. In that sense, the geometric term $E_{G}$ is given by

(2) $$E_{G}\left(T\right)=\sum_{(p,q)\in K}{\left(\left(p-Tq\right)\cdot n_{p}\right)}^{2}$$

where K is the correspondence set in the current iteration and $n_{p}$ is the normal of point p.

Similarly, the color term $E_{C}$ is expressed by

(3) $$E_{C}\left(T\right)=\sum_{(p,q)\in K}{\left(C_{p}\left(f\left(Tq\right)\right)-C\left(q\right)\right)}^{2}$$

The rationale for these terms is to measure the difference between the color of the point *q* (denoted as C(q)) and the color of its projection on the tangent plane *p*.

The main contribution of this multi-scale approach to the method is that it progressively refines the alignment starting from a coarse scale and gradually moves it to a finer one, thereby ensuring a globally optimal solution while avoiding local minima.

Upon successful pairwise registration, all the registered pairs are linked together. This process effectively consolidates the registered point clouds from each individual camera perspective into one unified, globally consistent coordinate frame. The final holistic point cloud brings together the different views, offering a comprehensive 3D representation of the object of interest. Figure 5c shows a registered point cloud of an animal after registering all the views.

To further refine our 3D model, we employ DBSCAN clustering [15,17], which effectively removes small, potentially erroneous point clusters. The result of this is a well-segmented point cloud ready for surface reconstruction.

#### 3.3.4. Trait Extraction

The pipeline concludes with a 3D surface reconstruction of the registered point cloud, facilitated by the Poisson Surface Reconstruction algorithm. As the name suggests, the Poisson Surface Reconstruction method aims to reconstruct a surface from a set of oriented points by computing an implicit function *X* that is 1 inside the surface and 0 outside. The actual surface is then reconstructed by extracting an appropriate isosurface. This is achieved by solving the Poisson equation ΔX=∇V, where *V* is a vector field derived from the normals of the oriented points, Δ is the Laplacian operator, and ∇ is the gradient operator. This algorithm is designed to reconstruct a smooth, watertight surface from a disorganized collection of 3D points [18,19], enabling the system to extract any traits of interest. While our current application primarily focuses on measuring the volume and surface area of cattle, this stage of the pipeline is designed to be adaptable, accommodating the extraction of various phenotypes according to user specifications. The system’s flexibility here allows users to define and extract additional traits of interest, extending beyond the initial focus on volume and surface area. Thus, although we demonstrate the utility of this process through our specific measurements, the scanning system is broadly applicable and can be tailored to meet the diverse needs of its different users.
**Pipeline 2** Scanning Software Pipeline1:**Input**2:         *I*     All frames (RGBD + K)3:        *C*     Color images4:        *D*     Depth images5:        *K*     Camera intrinsic matrices associated with images6:        *T*     Initial guess from Apriltag extrinsic calibration7:**Output**8:        *M*   Reconstructed mesh9:        SA   Surface area10:     *V*     Volume11:**for all** $\{D_{i},C_{i},K_{i}\}\in I$ **do**12:    $D_{i}^{f}\leftarrow \mathrm{FlyingPixelFilter}\left(D_{i}\right)$▹ Eliminate flying pixels13:    $C_{i}^{m},D_{i}^{m}\leftarrow \mathrm{MaskR}-\mathrm{CNN}\left(C_{i},D_{i}^{f}\right)$        ▹ Generate masked depth and color14:    $PCD_{i}^{m}\leftarrow \pi \left(C_{i}^{m},D_{i}^{m},K_{i}\right)$    ▹ Backproject to create **filtered** point cloud15:    $PCD_{i}\leftarrow \mathrm{TSDF}\left(C_{i},D_{i}\right)$             ▹ Generate **unfiltered** point cloud16:    $PCD_{list}^{m}.\mathrm{add}\left(PCD_{i}^{m}\right)$    ▹ Append $PCD_{i}^{m}$ to **filtered** point clouds17:    $PCD_{list}.\mathrm{add}\left(PCD_{i}\right)$    ▹ Append $PCD_{i}$ to **unfiltered** point clouds18:**end for**19:$PCD_{all}^{m},PCD_{all}\leftarrow \mathrm{PairAlign}\left(PCD_{list}^{m},PCD_{list},T\right)$                ▹ Stitch point clouds20:$PCD_{all}^{m}\leftarrow \mathrm{CompOrientNormals}\left(PCD_{all}^{m}\right)$            ▹ For **filtered** point cloud21:$PCD_{all}^{c}\leftarrow \mathrm{DBSCAN}\left(PCD_{all}^{m}\right)$    ▹ Refine segmentation via Clustering22:$M\leftarrow \mathrm{Poisson}\left(PCD_{all}^{c}\right)$                                                   ▹ Reconstruct Mesh23:$SA,V\leftarrow \mathrm{ExtractSurfaceAreaVolume}\left(M\right)$

### 3.4. Justification for Choice of Algorithms

At this point, we have covered the entire scanning pipeline from the acquisition of the data to the reconstruction of the cattle surface. In this section, we will present the rationale behind each of the pipeline’s specific choices of algorithms.

- **Colored ICP for Registration**: In the realm of 3D point cloud registration, numerous methods have been developed, which can be broadly categorized into local and global approaches. Global methods, such as those described in [20,21], typically rely on extracting geometric features like Fast Point Feature Histograms (FPFHs) [22]. However, these features do not always guarantee the production of unique and expressive descriptors, significantly impacting the algorithm’s ability to converge to a desirable result. Additionally, global methods do not produce tight alignment results and are primarily used to initialize local methods. Local methods, on the other hand, depend on a good coarse alignment of the point clouds as initialization. When there is a reliable initial alignment, these methods can guarantee convergence [14,23,24]. Given that we have colored point clouds and a fiduciary cube for obtaining a reliable initial alignment, we opted to use the Colored ICP algorithm [14]. This choice is justified since this local method optimizes not only a geometric objective but also a photometric objective using a multi-scale strategy, consistently resulting in a tight alignment of our point clouds.
- **Mask R-CNN for Segmentation**: The unified architecture of Mask R-CNN allows for the accurate identification and localization of multiple objects within complex scenes, making it an ideal solution for tasks demanding a detailed understanding of visual data. Mask R-CNN achieves state-of-the-art accuracy on several benchmarks due to its ability to refine the object boundaries and accurately segment instances [9].
- **Poisson for Surface Reconstruction**: In the field of 3D surface reconstruction, various methods have been developed to generate surfaces from unorganized point clouds. These methods can be broadly classified into explicit and implicit approaches. Explicit methods typically involve connecting sample points with triangles, resulting in an exact interpolation of the sample points. However, these methods often struggle with noisy data and can lead to holes or non-manifold situations. In contrast, implicit methods aim to find a manifold surface that approximates the original set of points, resulting in a closed, watertight reconstruction. Among explicit methods, the alpha shapes method [25] is a notable example. It generalizes the concept of a convex hull by initially wrapping the set of points in a convex hull and then carving away parts to reveal the underlying surface. Although effective, it tends to produce non-smooth surfaces. Similarly, the ball pivoting algorithm [26] is related to the alpha shapes method and reconstructs the surface by rolling a 3D ball with a fixed radius along the points. When the ball touches three points without falling through, it forms a triangle. The algorithm continues to pivot from the edges of existing triangles, creating new triangles as it encounters suitable point triplets. Like alpha shapes, the ball pivoting algorithm often results in non-smooth surfaces, making it less suitable for tasks requiring high-quality reconstructions. On the other hand, the Poisson surface reconstruction algorithm constructs surfaces by solving a regularized optimization problem, ensuring smoothness and continuity. It is particularly known for its robustness to noisy and irregularly sampled point clouds, making it more suitable for generating high-quality meshes.

## 4. Experimental Results and Discussions

In this section, we present the experimental results to demonstrate the effectiveness of our proposed scanning system in terms of accuracy and throughput. The experiments are designed to highlight the following aspects:

(a) The impact of effective synchronization on the quality of the scanning results.
(b) The precision of our proposed scanning system in estimating volume and surface area, by varying object position and orientation, object sizes, lighting conditions, and the distance to the sensor.
(c) Tests on real cattle to evaluate the system’s performance in terms of both accuracy and scanning speed.

### 4.1. Sensor Synchronization

The primary objective of these experiments is to demonstrate the crucial role of ToF sensor synchronization in optimizing data quality. ToF sensors operate by projecting modulated near-IR illumination onto a scene, and in a multi-sensor configuration, the potential for signal interference is high if emissions are not carefully timed. In our setup, ten (10) ToF sensors were arranged pairwise along a rod, and two differently sized boxes were used as scanning targets. A synchronization strategy was implemented, introducing a sequential delay of 160 μs between each sensor’s activation.

**Figure 6:**
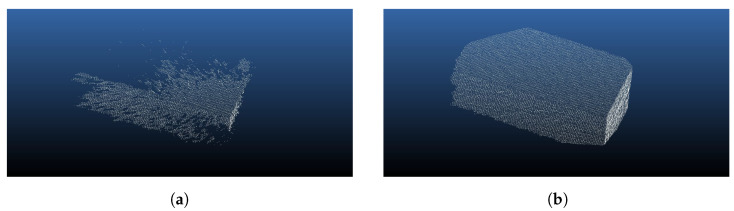
Comparison of 3D point cloud capture quality with and without synchronization using a large box with known dimensions. The left image displays the results without synchronization (0 μs), capturing a total of 17,098 points. The right image shows the same box captured with synchronization (160 μs) with all other settings the same, resulting in a total of 38,631 points, illustrating the significant improvement in data acquisition quality. (**a**) Large box, 0 s delay, *n* = 17,098. (**b**) Large box, 160 μs delay, *n* = 38,631.

We conducted scans in two distinct operational modes: with and without hardware synchronization, capturing images from ten different perspectives (N = 10). The qualitative impact of synchronization is profoundly demonstrated by the data: for instance, as shown in Figure 6, Camera 9 captured 38,631 points when synchronized; without synchronization, the point count drastically dropped to 17,098 for the same camera. Notably, as illustrated in Table 1, the master camera (Camera 1) returned a slightly higher point count when unsynchronized. This is expected since Camera 1 always fires first in both synchronized and unsynchronized capture modes, thus avoiding interference from other sensors. These results, illustrated in Table 1 and Figure 6, underscore that improper synchronization can lead to a significant loss of data—more than 50% of the object’s points in some cases. This stark contrast highlights the critical necessity of precise synchronization to prevent substantial data degradation and to ensure the reliability of multi-sensor ToF systems.

### 4.2. Experiments on Volume and Surface Area Accuracy Using Known Objects

Several experiments were conducted to validate the proposed scanning system’s accuracy in determining the target object’s volume and surface area. Four different objects, including three boxes and one cylinder with known dimensions, were used for testing.

#### 4.2.1. Single Object, Multiple Orientations

**Figure 7:**
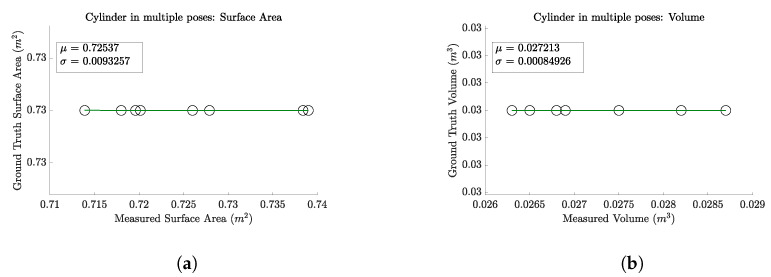
Results of scanning a cylindrical object in multiple orientations, highlighting the scanner’s accuracy across diverse poses. The horizontal axis displays the predicted volumes and surface areas obtained in each test. Given that the same object was used throughout, the ground truth volume and surface area remain constant. This plot demonstrates the scanner’s precision, as evidenced by the close alignment of the predicted values with the consistent ground truths, illustrating the system’s reliability in varying orientations. (**a**) Surface area calculation results. (**b**) Volume calculation results.

To demonstrate that the behavior of the scanning system is consistent with respect to the object’s pose, we conducted a test using a cylindrical object positioned in various configurations. We varied both the translation and orientation of the cylinder, placing it in eight (8) distinct positions. Images were captured from ten (10) different cameras for each position. The acquired RGB-D images were subsequently stitched together and then a mesh model of the cylinder was generated. We then calculated the surface area and volume based on this mesh. The results, presented in Figure 7, confirm that our scanner consistently and accurately computed the surface area and volume of the cylinder across all tested orientations. Specifically, the mean surface area was 0.725 m^2^ (averaged over eight poses), closely approaching the ground truth of 0.73 m^2^ with a narrow standard deviation of 0.009 m^2^. For volume, the mean value was 0.027 m^3^ (averaged over eight poses), nearly matching the ground truth of 0.03 m^3^ with an exceptionally low standard deviation of 0.0008 m^3^. These results not only affirm the precision of our measurements but also validate the robustness of our scanning system across diverse orientations.

#### 4.2.2. Multiple Objects, Static Pose

To further evaluate the performance of our scanning system, we conducted a series of tests focusing on its ability to consistently predict surface area and volume across a range of objects differing in size and shape. The test suite included a cylinder, a small box, a medium box, and a large box, each with precisely known dimensions. Given the demonstrated pose-independence of our scanner, all objects were placed in the same central position and subjected to 10 consecutive scans without altering their pose.

We computed the average predicted surface area and volume from these scans and performed a regression analysis to assess the precision of our measurements. The fit through the predicted volume and surface area data yielded an $R^{2}$ value of 0.997 for surface area and 0.999 for volume, indicating extremely high accuracy and consistency.

**Figure 8:**
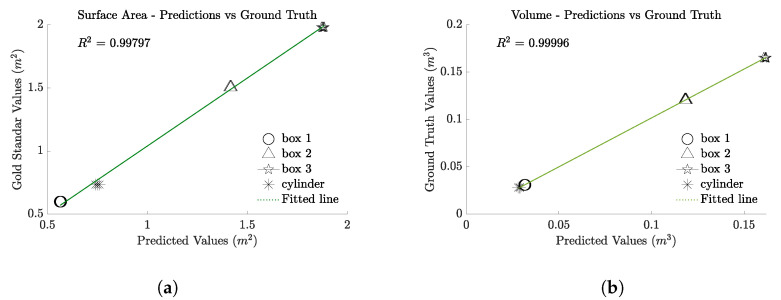
Regression analysis of predicted versus known surface area and volume for multiple static objects. The plots displays the correlation between the scanner’s predicted values and the actual measurements for a cylinder, small box, medium box, and large box, all placed in the same pose across 10 consecutive scans. The high $R^{2}$ values of 0.997 for surface area and 0.999 for volume demonstrate the scanner’s accuracy and consistency in various object dimensions and shapes under controlled conditions. (**a**) Surface area calculation results. (**b**) Volume calculation results.

The results, displayed in Figure 8, underscore the robustness and reliability of the scanner. The system not only predicts volume and surface area accurately across diverse object types but also demonstrates excellent repeatability. These findings confirm the efficacy of the scanner in various operational scenarios, enhancing its applicability in real-world conditions.

#### 4.2.3. Scanner Performance under Sunlight

To assess the robustness of our scanner in realistic operational environments, particularly for livestock (cattle) 3D scanning, we conducted an experiment in an outdoor setting under direct sunlight. This test aimed to determine the impact of sunlight on the accuracy of surface area and volume measurements. A single box was used for this purpose, with 10 consecutive scans performed to ensure consistent results.

The processed data from these scans were stitched together to create a mesh from which the surface area and volume were computed. The results indicate a mean volume of 0.10911 m^3^, with the ground truth being 0.125 m^3^, a 12.71% difference, with a standard deviation of 0.0090 m^3^. For surface area, the mean measurement across the scans was 1.393 m^2^, compared to the ground truth of 1.500 m^2^, a 7.13% difference with a standard deviation of 0.0999 m^2^.

**Figure 9:**
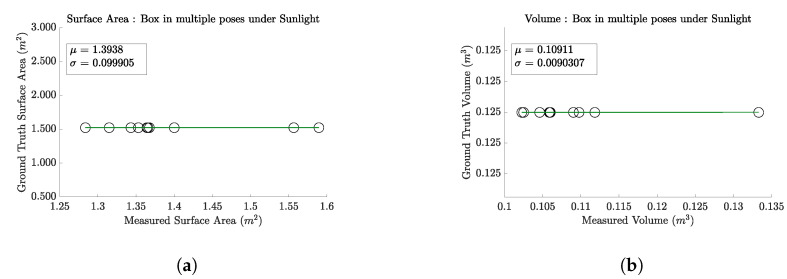
Performance of the scanner under direct sunlight, using a standard box to simulate outdoor livestock scanning conditions. The graphs show the mean and standard deviation of volume and surface area measurements across 10 consecutive scans. The results here illustrate the slight impact of sunlight on the scanner’s infrared sensors, affecting measurement accuracy. (**a**) Surface area calculation results from data collected in sunlight. (**b**) Volume calculation results from data collected in sunlight.

These findings, depicted in Figure 9, highlight the scanner’s ability to perform under challenging lighting conditions, though they also suggest a slight decrease in measurement accuracy due to the effects of sunlight on the infrared sensors. This insight is crucial for optimizing scanner deployment in outdoor livestock scanning scenarios.

#### 4.2.4. Varying Object Distance to the Sensor

In this experiment, we sought to explore how the distance of an object from the sensor influences the measured surface area and the number of points captured by the sensor. We positioned the object at incremental distances, starting from 2 feet up to 7 feet, from the sensor. Using a single ToF sensor, measurements were taken at each foot interval using both the narrow field of view (NFOV) and wide field of view (WFOV) settings of the ToF sensor.

The results, as depicted in Table 2, indicate that the surface area measurements and the number of points captured varied with distance, but not in a linear fashion. For both NFOV and WFOV, the maximum surface area was recorded at a distance of 3 feet with values of 0.349 m^2^ and 0.343 m^2^, respectively. Notably, both the surface area and the number of points tend to decrease as the object is positioned further from 3 feet, reaching their lowest at 7 feet with surface area values of 0.251 m^2^ for NFOV and 0.254 m^2^ for WFOV.

Interestingly, the measurements do not show a consistent decrease as distance increases, which suggests that the optimal distance for scanning in terms of maximizing the captured surface area and point density using our ToF sensor is around 3 feet. Beyond this point, both metrics begin to decline, possibly due to the spreading and weakening of the sensor’s signal with increased distance.

These findings highlight the importance of sensor placement relative to the target object in capturing the most accurate and detailed data possible.

### 4.3. Experiments on Real Cattle

Following the validation of the scanner on objects with known dimensions, we proceeded to conduct experiments on real cattle. The initial step in the experimental pipeline involved fine-tuning the segmentation network to adapt it specifically to cattle data.

#### Fine-Tuning Mask R-CNN

For this process, we captured both depth and RGB images using the camera sensors. Two pre-trained Mask R-CNN models were fine-tuned using these image types, respectively. To determine the most effective segmentation approach, we employed a voting arbitration mechanism between the two models. The outcomes of four different voting arbitration strategies are presented in Table 3.

The RGB-only model achieved the highest average Intersection over Union (IOU) score across all arbitrations, suggesting strong segmentation performance. However, this model also exhibited a high rate of false negatives, which could result in the loss of essential surface details crucial for accurate surface area computation of the cattle.

**Figure 10:**
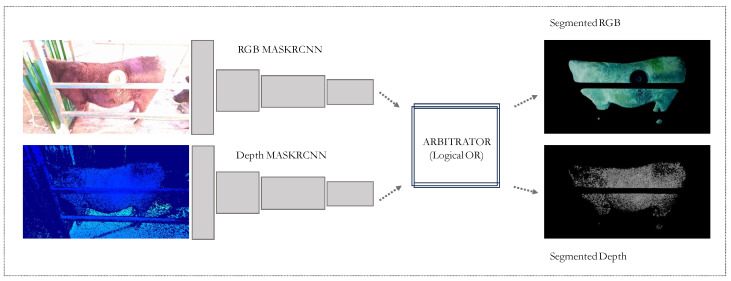
Segmentation of cattle using combined RGB and depth models via Mask R-CNN: The figure shows an RGBD image of cattle segmented using both RGB and depth data. Results from each model are integrated using a voting arbitrator, resulting in a well-defined segmentation in both modalities.

In contrast, the single-vote arbitration method, combining votes from both the depth and RGB models, provided an IOU score comparable to the RGB-only model but significantly reduced the false negative rate. Consequently, this method was selected for further segmentation tasks. Qualitative results of the segmentation on the test set are displayed in Figure 10.

After successfully detecting and masking the animals within the RGB and depth images, we used the generated masks to precisely extract the animals from the overall image. This segmentation isolates the animal alone, facilitating further processing of the data.

### 4.4. Extracting Surface and Volume Measurements on Cattle

**Figure 11:**
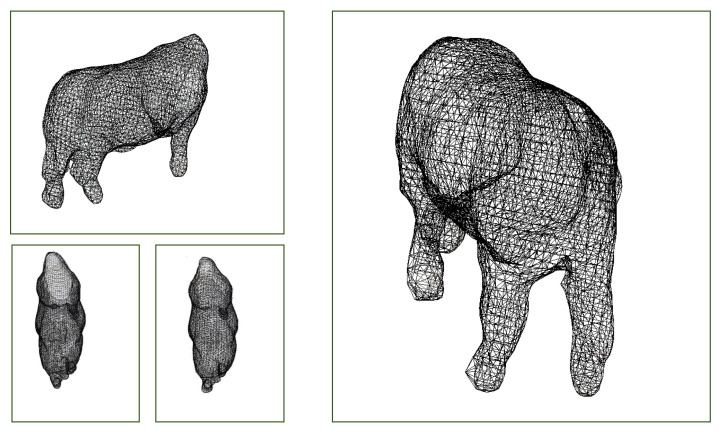
Poisson reconstructed meshes of cattle from which we compute the surface area and volume estimates.

Table 4 shows the statistics of the experiment conducted on live cattle versus their hides post-slaughter. Hides were removed at variable distances from the head and hooves. See Figure 11.

## 5. Conclusions and Future Works

This study introduced a new deep learning-based scanner optimized for high-throughput cattle phenotyping. Our proposed system proficiently captured and reconstructed meshes from various objects, demonstrating robustness across multiple challenging conditions. The key contributions of this work included the flexibility to use a variable number of time-of-flight sensors, the capability to scan untamed animals by effectively utilizing deep learning-based segmentation models to clean point clouds obtained from animals within a heavy-duty chute mechanism, and the full automation of the scanning pipeline, allowing researchers and farmers to scan large numbers of cattle with high throughput. Through a series of tests, we have shown that the scanner maintains consistent accuracy irrespective of the object’s geometry, orientation, position, distance to sensor, and exposure to environmental elements such as sunlight. In real-world applications, direct sunlight could be minimized using tents or other apparatus. The scanner has shown remarkable resilience in handling both stationary and untamed cattle, a capability enhanced by the our Server–Client program working in tandem with hardware synchronization. This synchronization effectively mitigated the issue of time-of-flight (ToF) sensor interference, which is crucial for accurate data acquisition.

The design of our system included a heavy-duty chute mechanism, which, together with timely camera triggers, guaranteed reliable data capture from dynamic subjects. The entire process, from capture to the computation of mesh measurements like volume and surface area, was completed within an average time of just 10.96 s. This efficiency underscored the system’s suitability for high-throughput applications, making it a valuable tool for modern agricultural practices. 

## Figures and Tables

**Table 1:** Measurements of the number of points generated in the synchronized and unsynchronized mode of the ToF sensor.

Sensor ID	Number of Points Synchronized (160 μs)	Number of Points Unsynchronized (0 μs)
Camera 1	33,607	36,062
Camera 2	16,127	15,235
Camera 3	15,333	13,937
Camera 4	24,743	22,969
Camera 5	28,597	25,573
Camera 6	32,044	27,303
Camera 7	42,803	29,432
Camera 8	37,469	35,046
Camera 9	37,583	35,149
Camera 10	38,631	17,098

**Table 2:** The statistical results of varying the distance of the object to the ToF sensor.

Distance to Sensor	Surface Area NFOV (m^2^)	Surface Area WFOV (m^2^)	Number of Points NFOV	Number of Points WFOV
2ft	0.290	0.294	2815	3058
3ft	0.349	0.343	3015	3253
4ft	0.278	0.280	2869	3135
5ft	0.315	0.335	3202	3442
6ft	0.277	0.283	2882	3153
7ft	0.251	0.254	2731	3010

**Table 3:** The statistical results (the average IOU, the false positive rate (FR), and the false negative rate (FN)) of using different voting arbitration for the segmentation models. The bold font specifies the best scores among the others.

Voting Arbitration	Avg. IOU	FP Rate (%)	FN Rate (%)
RGB only	**0.9653**	1.4480	3.7249
Depth only	0.9495	2.6401	4.9985
1-Vote (OR)	**0.9629**	3.2592	**2.2631**
2-Vote (AND)	0.9518	**0.7655**	6.4603

**Table 4:** Comparison between the measured surface area using the proposed 3D cattle scanner and the hand measurements from the hide of slaughtered animals. Each of the animals (which is determined by its cattle ID) was scanned 5 times and the average value was calculated over the scans.

Cattle ID	Manual-Measured Surface Area (m^2^)	Average Estimated Surface Area (m^2^)	Surface Area Std (m^2^)	Average Error (%)	Average Processing Time (s)
1	5.316	5.52270	0.09810	3.887	10.64
5	5.088	5.03896	0.09579	1.482	11.02
7	5.189	5.63482	0.08180	8.591	11.04
8	5.202	5.39062	0.06005	3.625	10.89
13	5.660	5.55332	0.12395	2.229	11.11
14	5.406	5.50881	0.04657	1.901	11.63
15	5.024	5.48532	0.05472	9.182	10.90
18	5.329	5.42638	0.09839	1.827	11.22
21	5.278	5.54710	0.06522	5.098	10.43
22	5.571	5.34380	0.15375	4.078	10.74
**Average**	-	-	**0.08783**	**4.1906**	**10.96**

## Data Availability

Data are contained within the article.
